# Phytochemicals From *Houttuynia cordata* Thunb as Potential Inhibitors of BRAF, MEK, and ERK: Insights From Molecular Docking

**DOI:** 10.1155/jskc/2565084

**Published:** 2025-11-28

**Authors:** Mongkol Yanarojana, Salunya Tancharoen, Thamthiwat Nararatwanchai, Somchai Yanarojana

**Affiliations:** ^1^School of Anti-Aging and Regenerative Medicine, Mae Fah Luang University, Bangkok, Thailand; ^2^Department of Pharmacology, Faculty of Dentistry, Mahidol University, Bangkok, Thailand; ^3^Department of Pharmacology, Faculty of Science, Mahidol University, Bangkok, Thailand

**Keywords:** BRAF, docking, ERK, hesperidin, *Houttuynia cordata* Thunb., melanoma, quercetin, RAF, rutin

## Abstract

This study utilized molecular docking techniques to investigate the potential of phytochemical compounds in *Houttuynia cordata* Thunb. extract as inhibitors of the oncogenic MAPK signaling pathway in melanoma. The docking results revealed that several phytochemical compounds exhibited favorable binding interactions with the BRAF^V600E^, MEK, and ERK ATP-binding site. A total of 16 compounds have high affinity (binding energies < −9 kcal/mol) for BRAF^V600E^, 13 compounds for MEK-1, 6 compounds for MEK-2, 18 compounds for ERK-1, and 10 compounds for ERK-2. Hesperidin exhibited the lowest binding energy to BRAF^V600E^ (−10.216 kcal/mol) and ERK-2 (−10.336 kcal/mol). Quercitrin has the lowest binding energy against MEK-1 (−9.963 kcal/mol), 3-hydroxy-β-sitost-5-en-7-one demonstrated the lowest binding energy to ERK-1 (−10.495 kcal/mol), and rutin was best against MEK-2 with a calculated binding energy value of −9.963 kcal/mol. The binding modes of these compounds are compared with the known inhibitors of the oncoprotein targets that showed similar interactions to key amino acid residues indicating their inhibitory potential and are suggested as promising candidates for melanoma treatment.

## 1. Introduction

Melanoma is a highly aggressive form of skin cancer that originates in melanocytes. Advanced stages of melanoma is characterized by its rapid proliferation, invasion, and metastasis and in many cases, it has been implicated to resistance to conventional therapeutics [1]. Many patients with metastatic forms of melanoma face poor prognosis with 1-year survival rates ranging from 33% to 62%, depending on the stage of the disease [2–4]. Activation of mutant RAS oncogenes is common in human cancers, and about 15%–20% of melanoma patients contain NRAS mutations, which are oncoproteins that activate BRAF signaling pathways [5]. To a greater extent, approximately 40%–60% of melanoma patients have BRAF mutations [6], and amongst these patients, 80%–90% exhibit a substitution of valine with glutamic acid at codon 600 (BRAF^V600E^) [6–8]. This mutation leads to the constitutive activation of the mitogen-activated protein kinase (MAPK) signaling pathway, resulting in uncontrolled cell growth and proliferation [9], thus BRAF mutants has been designated as a crucial drug target for the treatment of melanoma.

BRAF inhibitor drugs, such as dabrafenib and vemurafenib, inhibit BRAF mutants at the ATP binding site, which impedes BRAF signaling in the MAPK pathway, suppressing tumor growth [10–12]. However, resistance is common in monotherapy, necessitating combination treatments; combining BRAF and MEK inhibitors, such as trametinib and cobimetinib, has shown improvements in patient response and survival rates [13, 14]. Clinically approved MEK inhibitors target the allosteric site of MEK in the MAPK pathway. The combination use of BRAF and MEK inhibitors has become a standard first-line treatment for BRAF-mutant melanoma. However, these regimens have been conferred to resistance in tumors [15], and a need for new therapeutics is warranted. Recent advances include the development of ATP-competitive MEK and ERK inhibitors targeting the same MAPK pathway, which has shown potent growth inhibition of human tumors with resistance against standard BRAF and MEK inhibitors in vitro [16, 17] and in clinical trials [18]. So far, there are no drugs that selectively target ERKs and the ATP-binding site of MEK, which draws strong interest from researchers.

Plu-khao (*Houttuynia cordata* Thunb.) belongs to the Saururaceae family, also sometimes known as the “lizard's tail family,” native to North America, East and Southeast Asia. Plu-khao is a common plant used in traditional Thai cuisine, and due to its various pharmacological properties, including anticancer, antiallergy, antiviral, antibacterial, antioxidant, and anti-inflammatory properties, it is also used in traditional medicine [19]. Our previous in vitro study [20] revealed the antiproliferative effects and apoptosis-inducing properties of *Houttuynia cordata* Thunb. extracts against human A375 melanoma cells, which naturally harbor the BRAF^V600E^ oncogene [21]. This study takes a step further by employing molecular-docking simulations to investigate the potential inhibitory action of the MAPK signaling pathway and interactions of 74 phytochemical compounds from *Houttuynia cordata* Thunb. with BRAF, MEK, and ERK oncoproteins.

## 2. Materials and Methods

### 2.1. Retrieval and Preparation of Ligands for Molecular Docking

An extensive literature review was conducted to collect a comprehensive list of phytochemical compounds contained in *Houttuynia cordata* Thunb., as provided in Table S1 (Supporting Informations) [22]. Seventy-four phytochemical compounds were taken as ligands, and their chemical structures were downloaded in SDF file format from the PubChem database (https://pubchem.ncbi.nlm.nih.gov/) [23, 24]. Furthermore, ATP-competitive inhibitors were downloaded from the same source in SDF format and taken as ligands: a BRAF inhibitor drug, dabrafenib (CID: 44462760), a MEK inhibitor, MAP855 (CID: 90647159), and an ERK inhibitor, ulixertinib (CID: 11719003). The ligands were then prepared using the Open Babel tool (v 2.4.1) [25] of PyRx software (v1.1) [26] by minimizing their energies and converted into PDBQT file format for molecular docking simulations.

### 2.2. Retrieval and Preparation of Target Proteins for Molecular Docking

The three-dimensional (3D) structures of 5 target proteins were downloaded in PDB file format from the Protein Data Bank (PDB) [27]: BRAF^V600E^ (PDB ID: 3OG7 [10]), MEK-1 (PDB ID: 3SLS [28]), MEK-2 (PDB ID: 1S9I [29]), ERK-1 (PDB ID: 4QTB [30]), and ERK-2 (PDB ID: 6GDQ [31]). The target proteins were first prepared by removing solvents and cocrystallized ligands, addition of hydrogen atoms, partial charge adjustments, 3D protonation, and energy minimization using Discovery Studio Visualizer (Version 21.1.0.20290).

### 2.3. Molecular Docking Study

The PyRx with Vina Wizard was utilized in molecular docking simulations between 77 ligands and 5 target proteins to determine the binding affinities and ligand-protein interactions. The prepared structures of the target proteins were imported into PyRx and converted into PDBQT file format. An exhaustiveness value of 20 was used to maximize the ligand–protein binding conformational investigations. The best-docked conformations, characterized by the lowest docking scores and root mean square deviations (RMSDs), were selected for further analysis. Finally, Discovery Studio Visualizer was used to visualize the interactions between ligand and the target proteins.

## 3. Results and Discussion

**Figure 1:**
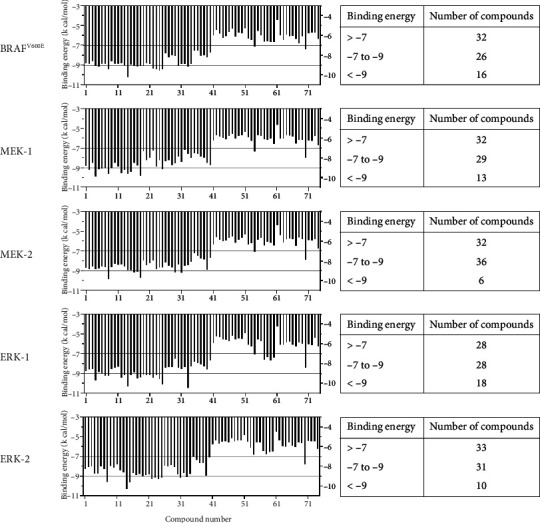
The binding energies of the phytochemical compounds with 5 target protein kinases: BRAF^V600E^, MEK-1, MEK-2, ERK-1, and ERK-2.

To predict the potency of phytochemical compounds in *Houttuynia cordata* Thunb. against the ATP binding sites of the kinases in MAPK signaling pathway for melanoma treatment, molecular docking simulations were performed using AutoDock Vina 1.2.5 [32]. The docking scores (binding energy) are shown in Table S2 (Supporting Information) and summarized for ease of illustration in a histogram shown in Figure 1. The binding energy ranges for the phytochemical compounds are as follows: −4.481–−10.216 kcal/mol for BRAF^V600E^, −4.667–−9.963 kcal/mol for MEK-1, −4.411–−9.917 kcal/mol for MEK-2, −4.318–−10.495 kcal/mol for ERK-1, and −4.517–−10.336 kcal/mol for ERK-2. The ligands were categorized into predicted weak inhibitors with binding energies > −7 kcal/mol, modest inhibitors with binding energies ranging from −7 to −9 kcal/mol and strong inhibitors with binding energies < −9 kcal/mol. In Figure 1, 16 compounds as strong inhibitors of BRAF^V600E^ mutant, 13 compounds as strong MEK-1 inhibitors, 6 compounds as strong MEK-2 inhibitors, 18 compounds as strong ERK-1, and 10 compounds as strong ERK-2 inhibitors; a tabulated list of phytochemical compounds with strong inhibition against the target proteins is provided in Table 1.

**Figure 2:**
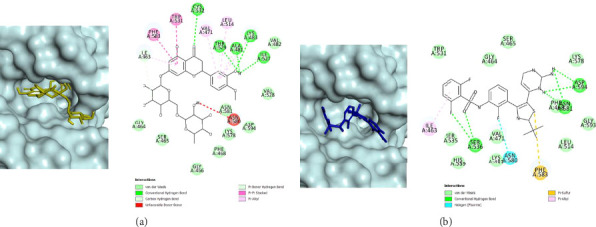
Predictive binding orientations using molecular docking simulations of hesperidin (a) and dabrafenib (b) against BRAF^V600E^. All ligands are bounded to the ATP binding site.

Against BRAF^V600E^ mutant, a total of 16 compounds were predicted to have strong inhibition including 6 flavonoid derivatives, all 4 aristolactams, 4 aporphines, and 2 steroid derivatives. It was predicted that the best inhibitor is hesperidin, a flavonoid derivative, that exhibited a calculated binding energy value of −10.216 kcal/mol, followed by two aporphines, 7-chloro-6-demethylcepharadione B, and noraristolodione with binding energy values of −9.589 and −9.490 kcal/mol, respectively. The calculated binding energy values of dabrafenib is −9.528 kcal/mol. Thus, hesperidin and 7-choloro-6-demethylcepharadione B were predicted to have binding energy values lower than dabrafenib, which indicates the two constituents are the most promising candidates. The predicted binding mode of hesperidin against BRAF^V600E^ mutant is shown in Figure 2(a) and for dabrafenib is shown in Figure 2(b). It was shown that the flavone moiety of hesperidin occupied the deep hydrophobic regions and formed π−π stacking interactions with Phe583 and Trp531, which was seen for other reported flavone-based scaffold inhibitors [33]. Hydrogen bonds were formed between the flavonoid phenol group and residues Thr529, Ala481, Lys483, Ile527, and Cys532. In comparison to dabrafenib, none of these residues formed hydrogen bonds with the drug indicating a distinctive mode of binding. Regardless, these interactions have been reported for other BRAF^V600E^ inhibitors from in silico screening [34]. A key hydrogen bond interaction is between dabrafenib and Asp594 which is part of the DFG motif that regulates the activation of BRAF by allowing ATP to form electrostatic interactions with the Mg^2+^ ion cofactor, a mechanism crucial for enzyme function [35, 36]; only nonpolar van der Waal's forces were seen between Asp594 and hesperidin. Other nonpolar van der Waal's forces were formed with residues Ile463, Gly464, Ser465, Gly466, Phe468, Val482, Val528, and Asn581; also, other hydrophobic π-interactions include with residues Val471 and Leu514, which has been observed for flavone-based inhibitors [33].

**Figure 3:**
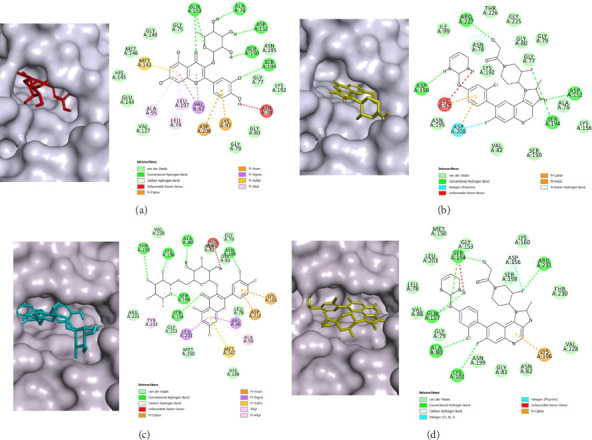
Predictive binding orientations using molecular docking simulations of quercitrin (a), MAP855 (b) against MEK-1, and the predicted binding orientations of rutin (c) and MAP855 (d) against MEK-2. All ligands are bounded to the ATP binding site.

Against MEK-1, 13 compounds were predicted to have strong inhibition: 11 flavonoid derivatives, piperolactam A from the aristolactam class, and noraristolodione from the aporphine class. The strongest inhibitors were predicted to be quercitrin, a flavonoid derivative that has binding energy value of −9.963 kcal/mol followed by piperolactam A with binding energy value of −9.844 kcal/mol and hesperidin that scored −9.668 kcal/mol. In comparison to the binding energy of the reference, MAP855 (−9.764 kcal/mol), quercitrin, and piperolactam A were predicted to have stronger binding to MEK-1 and potentially the most promising candidates. The predicted binding orientation of quercitrin is displayed in Figure 3(a). The hydrophobic flavone scaffold is inserted into the deep hydrophobic gorge of the ATP binding site, where nonpolar van der Waal's interactions were formed with residues His145, Glu144, Val127, Met146, Gly149, Gly75, Gly80, Gly79, Gly77, and Lys192. The aromatic flavone ring was predicted to form π-interactions with residues Met143, Asp208, Lys97, Val82, Leu197, Leu74, and Ala95. The π-interactions with residues Lys97 and Asp208 were also observed with MAP855, indicating the importance of these interactions for MEK-1 inhibition. Quercitrin were predicted to form hydrogen bonds with Glu153, Ala76, Asp152, Ser150, and Ser194. Residues Ser194 and Asp152 were seen to form hydrogen bonds with MAP855, indicating the importance of these interactions (Figure 3(b)). Hydrogen bond interactions with residues Ser150 and Ser194 have been observed for a known preclinical isoflavone-based compound and together with Asp152 for several drug candidates [37], indicating the medicinal potential of quercitrin as a MEK-1 inhibitor.

Six compounds were predicted to have strong inhibition against MEK-2 with rutin, a flavonoid derivative, predicted to be the strongest inhibitor, 3 aristolactams, procyanidin B1 which is a phenolic compound, and a steroid, β-sitosteryl glucoside. The top three candidates include rutin as the best candidate with a binding energy value of −9.963 kcal/mol, piperolactam A with a binding energy value of −9.712 kcal/mol, and aristolactam BII that scored −9.196 kcal/mol; all candidates have binding energy values lower than the reference control, MAP855, that scored −8.997 kcal/mol. The predicted binding orientation of rutin is shown in Figure 3(c). The hydrophobic flavone scaffold is bound to the deep hydrophobic regions of the ATP binding site where van der Waal's interactions are favorable with residues Val228, Leu78, Gly79, Gly81, His149, Met150, Gly153, Arg237, and Asn82. Hydrogen bonds were observed between the ketone of flavone and Ser154. Evidently, this interaction was also observed with MAP855 (Figure 3(d)), in which Ser154 formed a hydrogen bond interaction with oxygen functionalities such as ether and alcohol. Additionally, hydrogen bonds were formed between the sugar moieties and residues Ser198, Asn199, Ala80, Lys196, and Thr230. Hydrophobic π-interactions that include aromatic systems were formed with residues Leu201, Val86, Ala99, Tyr233, Lys101, Asp212, and Met147. Several of these interaction have been reported with previous studies which have identified flavonoid derivatives as inhibitors of MEK-2 [38], indicating the plausibility of rutin as a potential MEK-2 inhibitor.

**Figure 4:**
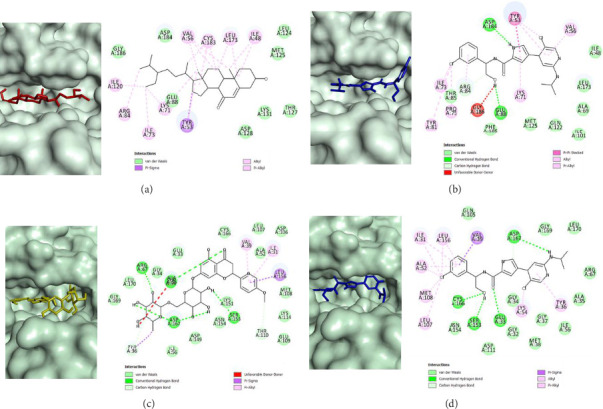
Predictive binding orientations using molecular docking simulations of 3-hydroxy-β-sitost-5-en-7-one (a), ulixertinib (b) against ERK-1, and the predicted binding orientations of hesperidin (c) and ulixertinib (d) against ERK-2. All ligands are bounded to the ATP binding site.

ERKs are proteins that contribute to downstream MAPK signaling. So far, there are no drugs approved that selectively target ERKs. Ulixertinib, a drug candidate which was tested for its use in metastatic uveal melanoma treatment was used as the control [39]. Ulixertinib displayed *K*_*i*_ values of 0.3 and 0.04 nmol/L for ERK-1 and ERK-2, respectively, and has shown significant reduction in tumor volume A375 and COLO205 cell line xenografts, with it being classified as an ATP-competitive inhibitor [40]. Eighteen compounds have been identified as potentially potent ERK-1 inhibitors which include 8 flavonoid derivatives, 3 aristolactams, 6 aporphines, and 3-hydroxy-β-sitost-5-en-7-one steroid derivative, which was predicted to be the most potent with binding energy value of −10.495 kcal/mol. Minimal biological activities were reported for this specific compound, but it has been tested as part of extract mixtures that contain cytotoxic activities [41, 42]. The second most potent inhibitor was predicted to be the quercitrin with binding energy value of −9.735 kcal/mol followed by an aporphine, 7-chloro-6-demethylcepharadione B, with binding energy value of −9.616 kcal/mol. A known ERK-1 inhibitor, ulixertinib, was used as the reference control which scored −9.951 kcal/mol; only 3-hydroxy-β-sitost-5-en-7-one displayed a lower binding energy suggesting it to be the most promising candidate. The binding orientation of 3-hydroxy-β-sitost-5-en-7-one to the ATP binding site of ERK-1 is shown in Figure 4(a); the compound is mainly hydrophobic, and thus, it was predicted that mainly hydrophobic interactions contributed to ERK-1 binding as seen for nonpolar van der Waal's interactions with residues Asp184, Gly186, Glu88, Asp128, Lys131, Thr127, Met125, and Leu124. Other hydrophobic interactions were observed with mainly hydrophobic amino acid residues and nonpolar side chains of Tyr53, Ile120, Ile73, Lys71, Val56, Cys183, Leu173, and Ile48. The reference control, ulixertinib, formed hydrogen bonds with Glu88 and Asp184, which are key residues for ERK-1 inhibition (Figure 4(b)); 3-hydroxy-β-sitost-5-en-7-one formed nonpolar van der Waal's interactions with these residues, indicating possible strong inhibition with ERK-1.

Against ERK-2, 10 compounds were predicted to have strong affinity, which includes 3 flavonoid derivatives, caldensine which is an aristolactam, 4 aporphines, and 2 steroid derivatives. The strongest inhibitor predicted was hesperidin with a binding energy value of −10.336 kcal/mol, followed by the flavonoid derivative genistin and rutin that scored −9.672 and −9.605 kcal/mol, respectively. The predicted binding mode of hesperidin to the ATP binding site of ERK-2 is shown in Figure 4(c). The hydrophobic flavone core scaffold is bounded to the deep hydrophobic pocket of the binding site where it formed nonpolar van der Waal's interactions with Cys166, Leu107, Asp106, Ala52, Met108, Lys114, Thr110, Glu109, and Lys151, whereas the sugar side chains formed the same interactions with Asn154, Asp149, Ile56, Tyr36, Gly169, Leu170, Gly34, and Glu33. Hydrogen bond interactions were formed between the flavone ketone and Ala35 residue, whereas the hydroxyl groups on the sugar side chains formed hydrogen bond interactions with Arg67, Asp167, and Ser153. Hydrogen bond interactions with Asp167 and Ser153 were also observed with ulixertinib, in which these residues have been reported as important for ERK-2 inhibition (Figure 4(d)) [43, 44]. Other hydrophobic interactions include π-interactions between the phenyl group and residues Leu156, Ile31, and Val39.

Based on the simulation results, certain phytochemical constituents are suggested to have potential as strong inhibitors of the enzymatic targets in the MAPK signaling pathway and can serve as new candidates for the treatment of melanoma. Some compounds scored very strongly against multiple targets in the pathway. For example, hesperidin was suggested as strong inhibitors against BRAF^V600E^, MEK-2, and ERK-2, which is in line with reports of hesperidin being able to attenuate phosphorylation of MEK5 and ERK5 in cholangiocarcinoma cells [45], and suppression of MAPK signaling in prostate cancer resulting in reduction of ERK activities [46]. In this study, quercitrin was suggested as a strong inhibitor of both MEK-1 and ERK-1, which is supported by its ability to induce apoptosis in gastric cancer cells through suppression of EGFR-ERK signaling pathway [47] and has been suggested to inhibit MEK-1 activities, resulting in inhibition of TPA-induced neoplastic transformation of JB6 P+ cells [48]. The aporphine compound, 7-chloro-6-demethylcepharadione B, has not been reported to show effects against MAPK signaling, but in this study, it scored strongly against both BRAF^V600E^ and ERK-1. The aristolactam compound, piperolactam A, has been suggested as adequate inhibitors against MEK-1 and MEK-2. This finding is consistent with reports of extracts containing piperolactam A from *Piper* plants that demonstrated suppression of MEK/ERK signals resulting in colon cancer cell death [49, 50].

## 4. Conclusions

In this study, several phytochemical compounds from *Houttuynia cordata* Thunb. extract were identified as potential inhibitors of kinase targets in the MAPK signaling pathway and may serve as plausible candidates for the treatment of melanoma. Hesperidin scored best against BRAF^V600E^ and showed strong inhibitory action potential against MEK-2 and ERK-2. Quercitrin was predicted to be the best ATP-competitive inhibitor candidate for MEK-1 inhibition, whilst also displayed strong potential against ERK-1. Rutin was predicted to be the best ATP-competitive MEK-2 inhibitor candidate with predicted strong binding to ERK-2. The steroid derivative, 3-hydroxy-β-sitost-5-en-7-one, was identified to potentially be the best candidate against ERK-1, though a lack experimental evidence and scarce literature support undermined the suggestion with the same reason for it toward BRAF^V600E^, which was implied to be another potential target in the signaling pathway. This work emphasizes the effectiveness of molecular docking as a technique for the identification of plausible compound candidates in plant-based therapies for melanoma treatment.

## Figures and Tables

**Table 1:** Binding energies of phytochemical compounds with predicted strong inhibition against 5 target protein kinases.

Compound number	Compound class	Compound name	Binding energy (kcal/mol)				
			BRAF^V600E^	MEK-1	MEK-2	ERK-1	ERK-2
1	Flavonoid	Luteolin	−8.842	−8.889	−8.759	−8.763	−8.321
2	Flavonoid	Quercetin	−8.873	−9.271	−8.854	−8.600	−8.110
3	Flavonoid	Isorhamnetin	−8.597	−8.555	−8.610	−8.550	−8.059
4	Flavonoid	Quercitrin	−9.081	−9.963^a^	−8.888	−9.735	−8.750
5	Flavonoid	Isoquercitrin	−9.187	−9.165	−8.805	−8.882	−8.736
6	Flavonoid	Hyperin	−8.904	−9.130	−8.590	−9.042	−8.047
7	Flavonoid	Avicularin	−8.888	−9.066	−8.653	−9.262	−8.342
8	Flavonoid	Rutin	−9.435	−9.658	−9.917^a^	−9.256	−9.605
9	Flavonoid	Catechin	−8.674	−9.052	−8.670	−8.538	−8.005
10	Flavonoid	Apigenin	−8.865	−8.540	−8.311	−8.465	−8.167
11	Flavonoid	Kaempferol	−8.896	−8.923	−8.456	−8.369	−7.821
12	Flavonoid	Afzelin	−8.846	−9.564	−8.380	−9.457	−8.407
13	Flavonoid	Phlorizin	−9.066	−9.248	−8.594	−9.203	−8.652
14	Flavonoid	Hesperidin	−10.216^a^	−9.668	−8.987	−10.365	−10.336^a^
15	Flavonoid	Genistin	−9.014	−9.442	−8.960	−9.231	−9.672
16	Aristolactam	Aristolactam BII	−9.086	−8.537	−9.196	−8.928	−8.704
17	Aristolactam	Aristolactam AII	−9.184	−8.826	−9.144	−9.543	−8.904
18	Aristolactam	Piperolactam A	−9.100	−9.844	−9.712	−9.417	−8.788
19	Aristolactam	Caldensine	−9.113	−7.356	−7.986	−9.178	−9.019
20	Aporphine	Splendidine	−8.822	−8.286	−8.493	−9.219	−8.910
21	Aporphine	Lysicamine	−8.936	−8.001	−8.290	−9.191	−8.870
22	Aporphine	Cepharadione B	−9.389	−7.317	−7.976	−9.494	−9.273
23	Aporphine	Norcepharadione B	−9.471	−8.918	−8.844	−9.214	−9.181
24	Aporphine	7-Chloro-6-demethylcepha radione B	−9.589	−8.250	−8.711	−9.616	−9.323
25	Aporphine	Noraristolodione	−9.490	−9.179	−8.729	−10.112	−9.218
27	Phenolic	Neochlorogenic acid	−8.242	−8.281	−8.524	−8.408	−8.027
29	Phenolic	Procyanidin B1	−8.140	−8.572	−9.170	−7.532	−8.126
30	Steroid	β-Sitosterol	−9.084	−7.903	−8.359	−8.442	−8.883
31	Steroid	β-Sitosteryl glucoside	−8.925	−8.455	−9.232	−8.554	−9.208
32	Steroid	5-α-Stigmastane-3,6-dione	−8.942	−7.257	−8.547	−8.421	−8.690
33	Steroid	3-Hydroxy-β-sitost-5-en-7-one	−9.233	−7.615	−8.497	−10.495^a^	−9.126
34	Triterpenoid	Cycloart-25-ene-3,24-diol	−8.954	−8.059	−8.106	−8.304	−8.821
36	Benzamide	N-(4-hydroxyphenylethyl) benzamide	−7.559	−7.595	−7.496	−7.955	−7.387
75	—	Dabrafenib	−9.528				
76	—	MAP855		−9.764	−8.997		
77	—	Ulixertinib				−9.951	−9.361

^a^Phytochemicals with strongest inhibition properties.

## Data Availability

Data are contained within the article and Supporting Informations.

## Supporting Information

Supporting Table S1: List of controls and phytochemicals in *Houttuynia cordata* Thunb.

Table S2: Calculated binding energy values.
