# Supplementary material for: Phytochemicals From Houttuynia cordata Thunb as Potential Inhibitors of BRAF, MEK, and ERK: Insights From Molecular Docking
Source: J Skin Cancer. 2025 Nov 28;2025:2565084. doi: 10.1155/jskc/2565084 (PMC12680463; doi:10.1155/jskc/2565084)
Supplement: Supporting Information — Additional supporting information can be found online in the Supporting Information section. [file 2565084.f1.docx]

**Table S1**. List of controls and phytochemicals in *Houttuynia cordata* Thunb.

| No. | Name | PubChem CID  Chemical structure |
| --- | --- | --- |
| 1 | Luteolin  3′,4′,5,7-Tetrahydroxyflavone | 5280445   |
| 2 | Quercetin  3,3′,4′,5,7-Pentahydroxyflavone | 5280343   |
| 3 | Isorhamnetin / 3-Methylquercetin /  Quercetin 3'-methyl ether  3,5,7-trihydroxy-2-(4-hydroxy-3-methoxyphenyl)-4H-chromen-4-one | 5281654   |
| 4 | Quercitrin /  Quercetin 3-rhamnoside  3′,4′,5,7-Tetrahydroxy-3-(α-L-rhamnopyranosyloxy) flavone | 5280459   |
| 5 | Isoquercitrin  3-(β-D-Glucopyranosyloxy)-3′,4′,5,7-tetrahydroxyflavone | 5280804   |
| 6 | Hyperin / Hyperoside /  Quercetin 3-galactoside  3-(β-D-Galactopyranosyloxy)-3′,4′,5,7-tetrahydroxyflavone | 5281643   |

**Table S1**. List of phytochemicals in *Houttuynia cordata* Thunb. (Cont.)

| 7 | Avicularin  3-(((2R,3S,4S,5R)-3,4-dihydroxy-5-(hydroxymethyl)tetrahydrofuran-2-yl)oxy)-2-(3,4-dihydroxyphenyl)-5,7-dihydroxy-4H-chromen-4-one | 5490064   |
| --- | --- | --- |
| 8 | Rutin / Quercetin 3-rutinoside  3′,4′,5,7-Tetrahydroxy-3-[α-L-rhamnopyranosyl-(1→6)-β-D-glucopyranosyloxy]flavone | 5280805   |
| 9 | Catechin  (2R,3S)-2-(3,4-dihydroxyphenyl)chromane-3,5,7-triol | 9064   |
| 10 | Apigenin  4′,5,7-Trihydroxyflavone | 5280443   |
| 11 | Kaempferol  3,5,7-trihydroxy-2-(4-hydroxyphenyl)-4H-chromen-4-one | 5280863   |
| 12 | Afzelin / Kaempferol 3-rhamnoside  4′,5,7-Trihydroxy-3-(α-D-rhamnopyranosyloxy)flavone | 5316673   |

**Table S1**. List of phytochemicals in *Houttuynia cordata* Thunb. (Cont.)

| 13 | Phlorizin | 6072   |
| --- | --- | --- |
| 14 | Hesperidin  (2S)-3′,5-Dihydroxy-4′-methoxy-7-[α-L-rhamnopyranosyl-(1→6)-β-D-glucopyranosyloxy]flavan-4-one | 10621   |
| 15 | Genistin | 5281377   |
| 16 | Aristolactam BII / Cepharanone B  1,2-dimethoxydibenzo[cd,f]indol-4(5H)-one | 162739   |
| 17 | Aristolactam AII  2-hydroxy-1-methoxydibenzo[cd,f]indol-4(5H)-one | 148657   |
| 18 | Piperolactam A / Aristolactam F1  1-hydroxy-2-methoxydibenzo[cd,f]indol-4(5H)-one | 3081016   |
| 19 | Caldensine  1,2-dimethoxy-5-methyldibenzo[cd,f]indol-4(5H)-one | 21680139   |

**Table S1**. List of phytochemicals in *Houttuynia cordata* Thunb. (Cont.)

| 20 | Splendidine  1,2,4-trimethoxy-7H-dibenzo[de,g]quinolin-7-one | 196452   |
| --- | --- | --- |
| 21 | Lysicamine / Oxonuciferine  1,2-dimethoxy-7H-dibenzo[de,g]quinolin-7-one | 122691   |
| 22 | Cepharadione B  1,2-dimethoxy-6-methyl-4H-dibenzo[de,g]quinoline-4,5(6H)-dione | 189151   |
| 23 | Norcepharadione B  1,2-dimethoxy-4H-dibenzo[de,g]quinoline-4,5(6H)-dione | 189168   |
| 24 | 7-Chloro-6-demethylcepharadione B  7-chloro-1,2-dimethoxy-4H-dibenzo[de,g]quinoline-4,5(6H)-dione | 131752718   |
| 25 | Noraristolodione  2-hydroxy-1-methoxy-4H-dibenzo[de,g]quinoline-4,5(6H)-dione | 10108434   |

**Table S1**. List of phytochemicals in *Houttuynia cordata* Thunb. (Cont.)

| 26 | Chlorogenic Acid  (1S,3R,4R,5R)-3-(((E)-3-(3,4-dihydroxyphenyl)acryloyl)oxy)-1,4,5-trihydroxycyclohexane-1-carboxylic acid | 1794427   |
| --- | --- | --- |
| 27 | Neochlorogenic acid  (1R,3R,4S,5R)-3-(((E)-3-(3,4-dihydroxyphenyl)acryloyl)oxy)-1,4,5-trihydroxycyclohexane-1-carboxylic acid | 5280633   |
| 28 | Cryptochlorogenic acid  (3R,5R)-4-(((E)-3-(3,4-dihydroxyphenyl)acryloyl)oxy)-1,3,5-trihydroxycyclohexane-1-carboxylic acid | 9798666   |
| 29 | Procyanidin B1  (2R,2'R,3R,3'S,4R)-2,2'-bis(3,4-dihydroxyphenyl)-[4,8'-bichromane]-3,3',5,5',7,7'-hexaol | 11250133   |
| 30 | β-Sitosterol / Stigmast-5-en-3β-ol | 222284 |
| 31 | β-Sitosteryl glucoside | 5742590 |

**Table S1**. List of phytochemicals in *Houttuynia cordata* Thunb. (Cont.)

| 32 | 5-α-Stigmastane-3,6-dione | 13992092 |
| --- | --- | --- |
| 33 | 3-Hydroxy-β-sitost-5-en-7-one | 160608 |
| 34 | Cycloart-25-ene-3,24-diol | 11419367 |
| 35 | N-(1-hydroxy-3-phenylpropan-2-yl)benzamide | 100005   |
| 36 | N‑(4‑hydroxyphenylethyl)  benzamide | 433864354   |
| 37 | trans-N‑(4‑hydroxystyryl)  benzamide | 5369805   |
| 38 | Houttuynamide A | 44521377   |

**Table S1**. List of phytochemicals in *Houttuynia cordata* Thunb. (Cont.)

| 39 | Houttuynoside A | 44521323 |
| --- | --- | --- |
| 40 | 6,7-dimethyl-1-(2,4,5-trihydroxy-3-methylpentyl)-1,4-dihydroquinoxaline-2,3-dione | 605462   |
| 41 | 4-Hydroxyquinoline | 69141   |
| 42 | Benzamide / Phenylcarboxyamide | 2331   |
| 43 | 4-Hydroxybenzamide | 65052   |
| 44 | 4-Hydroxy-3-methoxybenzamide | 354088   |
| 45 | Vanillic acid | 8468   |
| 46 | Methyl vanillate | 19844   |

**Table S1**. List of phytochemicals in *Houttuynia cordata* Thunb. (Cont.)

| 47 | Vanillin | 1183   |
| --- | --- | --- |
| 48 | Protocatehuic acid  3,4-dihydroxybenzoic acid | 72   |
| 49 | 4-Hydroxybenzoic acid | 135   |
| 50 | Methylparaben | 7456   |
| 51 | p-Hydroxybenzaldehyde | 126   |
| 52 | Methyl cis-ferulate | 10176654   |
| 53 | Methyl trans-ferulate | 5357283   |
| 54 | Benzyl-β-D-glucopyranoside | 13254166   |
| 55 | Methyl 3-hydroxybenzoate | 88068   |

**Table S1**. List of phytochemicals in *Houttuynia cordata* Thunb. (Cont.)

| 56 | Methyl 4-(hydroxymethyl) benzoate | 81325   |
| --- | --- | --- |
| 57 | 1,3,5-Tridecanoylbenzene | 86173717   |
| 58 | 3,5-Didecanoylpyridine | 85697557   |
| 59 | 5-Decanoyl-2-nonylpyridine | 85697559   |
| 60 | 3,5-didecanoyl-4-nonyl-1,4-dihydropyridine | 129711227   |
| 61 | 5-Methoxy-1-methylpyrrolidin-2-one | 11423602   |
| 62 | 3-Nonyl-1H-pyrazole | 24844218   |
| 63 | Myristicin | 4276   |
| 64 | Elemicin | 10248   |

**Table S1**. List of phytochemicals in *Houttuynia cordata* Thunb. (Cont.)

| 65 | 4-allyl-2,6-dimethoxyphenol | 226486   |
| --- | --- | --- |
| 66 | α-Asarone | 636822   |
| 67 | Indole-3-carboxylic acid | 69867   |
| 68 | Vomifoliol | 5280462   |
| 69 | Dehydrovomifoliol | 688492   |
| 70 | Roseoside | 73815023   |
| 71 | (E)-1-(3-hydroxybut-1-en-1-yl)-2,6,6-trimethylcyclohexane-1,2,4-triol | 72751004   |
| 72 | (E)-4-(1,2,4-trihydroxy-2,6,6-trimethylcyclohexyl)but-3-en-2-one | 51136538   |

**Table S1**. List of phytochemicals in *Houttuynia cordata* Thunb. (Cont.)

| 73 | Quinic acid | 6508   |
| --- | --- | --- |
| 74 | Caffeic Acid | 689043   |
| 75 | Dabrafenib | 44462760   |
| 76 | MAP855 | 90647159   |
| 77 | Ulixertinib | 11719003   |

**Table S2.** Binding energy (kcal/mol) of compounds docked with their target proteins.

| No. | Binding energy (kcal/mol) | | | | |
| --- | --- | --- | --- | --- | --- |
|  | BRAF^V600E^ | MEK-1 | MEK-2 | ERK-1 | ERK-2 |
| 1 | -8.842 | -8.889 | -8.759 | -8.763 | -8.321 |
| 2 | -8.873 | -9.271 | -8.854 | -8.600 | -8.110 |
| 3 | -8.597 | -8.555 | -8.610 | -8.550 | -8.059 |
| 4 | -9.081 | -9.963 | -8.888 | -9.735 | -8.750 |
| 5 | -9.187 | -9.165 | -8.805 | -8.882 | -8.736 |
| 6 | -8.904 | -9.130 | -8.590 | -9.042 | -8.047 |
| 7 | -8.888 | -9.066 | -8.653 | -9.262 | -8.342 |
| 8 | -9.435 | -9.658 | -9.917 | -9.256 | -9.605 |
| 9 | -8.674 | -9.052 | -8.670 | -8.538 | -8.005 |
| 10 | -8.865 | -8.540 | -8.311 | -8.465 | -8.167 |
| 11 | -8.896 | -8.923 | -8.456 | -8.369 | -7.821 |
| 12 | -8.846 | -9.564 | -8.380 | -9.457 | -8.407 |
| 13 | -9.066 | -9.248 | -8.594 | -9.203 | -8.652 |
| 14 | -10.216 | -9.668 | -8.987 | -10.365 | -10.336 |
| 15 | -9.014 | -9.442 | -8.960 | -9.231 | -9.672 |
| 16 | -9.086 | -8.537 | -9.196 | -8.928 | -8.704 |
| 17 | -9.184 | -8.826 | -9.144 | -9.543 | -8.904 |
| 18 | -9.100 | -9.844 | -9.712 | -9.417 | -8.788 |
| 19 | -9.113 | -7.356 | -7.986 | -9.178 | -9.019 |
| 20 | -8.822 | -8.286 | -8.493 | -9.219 | -8.910 |
| 21 | -8.936 | -8.001 | -8.290 | -9.191 | -8.870 |
| 22 | -9.389 | -7.317 | -7.976 | -9.494 | -9.273 |
| 23 | -9.471 | -8.918 | -8.844 | -9.214 | -9.181 |
| 24 | -9.589 | -8.250 | -8.711 | -9.616 | -9.323 |
| 25 | -9.490 | -9.179 | -8.729 | -10.112 | -9.218 |
| 26 | -7.848 | -8.356 | -8.224 | -8.436 | -7.971 |
| 27 | -8.242 | -8.281 | -8.524 | -8.408 | -8.027 |
| 28 | -8.046 | -8.757 | -8.712 | -8.379 | -7.892 |
| 29 | -8.140 | -8.572 | -9.170 | -7.532 | -8.126 |
| 30 | -9.084 | -7.903 | -8.359 | -8.442 | -8.883 |
| 31 | -8.925 | -8.455 | -9.232 | -8.554 | -9.208 |
| 32 | -8.942 | -7.257 | -8.547 | -8.421 | -8.690 |
| 33 | -9.233 | -7.615 | -8.497 | -10.495 | -9.126 |
| 34 | -8.954 | -8.059 | -8.106 | -8.304 | -8.821 |
| 35 | -7.610 | -7.574 | -7.226 | -7.858 | -7.068 |
| 36 | -7.559 | -7.595 | -7.496 | -7.955 | -7.387 |
| 37 | -8.063 | -7.873 | -7.765 | -8.139 | -7.685 |
| 38 | -8.021 | -8.005 | -7.888 | -8.287 | -7.686 |
| 39 | -8.222 | -8.560 | -8.902 | -8.631 | -8.960 |
| 40 | -7.730 | -8.762 | -7.735 | -7.689 | -7.124 |
| 41 | -6.039 | -6.239 | -6.365 | -5.964 | -5.840 |
| 42 | -5.481 | -5.704 | -5.612 | -5.238 | -5.417 |
| 43 | -5.859 | -5.880 | -5.893 | -5.362 | -5.707 |
| 44 | -6.313 | -5.989 | -6.037 | -5.561 | -5.543 |
| 45 | -6.150 | -6.130 | -5.973 | -5.649 | -5.515 |
| 46 | -5.705 | -5.844 | -5.704 | -5.767 | -5.585 |
| 47 | -5.373 | -5.620 | -5.534 | -5.187 | -5.197 |
| 48 | -6.187 | -6.086 | -6.185 | -5.562 | -5.483 |
| 49 | -5.760 | -5.844 | -5.898 | -5.464 | -5.409 |
| 50 | -5.807 | -5.731 | -5.692 | -5.523 | -5.397 |
| 51 | -5.296 | -5.399 | -5.318 | -4.948 | -4.868 |
| 52 | -6.329 | -5.943 | -6.373 | -6.153 | -5.567 |
| 53 | -6.527 | -6.318 | -6.264 | -6.282 | -6.125 |
| 54 | -7.171 | -7.406 | -7.113 | -7.096 | -6.865 |
| 55 | -5.612 | -5.694 | -5.856 | -5.566 | -5.606 |
| 56 | -6.005 | -5.822 | -5.706 | -5.744 | -5.635 |
| 57 | -6.660 | -6.160 | -6.477 | -7.639 | -6.493 |
| 58 | -6.600 | -6.202 | -6.105 | -7.355 | -6.820 |
| 59 | -6.699 | -6.102 | -6.190 | -7.700 | -6.565 |
| 60 | -6.719 | -6.630 | -6.465 | -7.444 | -6.526 |
| 61 | -4.481 | -4.667 | -4.411 | -4.318 | -4.517 |
| 62 | -6.010 | -6.094 | -5.427 | -6.125 | -5.409 |
| 63 | -6.504 | -6.088 | -6.129 | -6.145 | -5.992 |
| 64 | -5.975 | -5.719 | -5.828 | -5.937 | -6.058 |
| 65 | -6.130 | -5.773 | -5.779 | -5.829 | -5.592 |
| 66 | -6.097 | -5.872 | -5.827 | -6.111 | -5.919 |
| 67 | -6.423 | -6.600 | -6.554 | -6.279 | -6.110 |
| 68 | -6.860 | -6.195 | -5.684 | -5.906 | -5.599 |
| 69 | -6.113 | -6.200 | -5.836 | -6.043 | -5.651 |
| 70 | -7.438 | -8.015 | -7.951 | -8.467 | -7.818 |
| 71 | -5.779 | -6.269 | -5.925 | -6.067 | -5.437 |
| 72 | -5.768 | -6.324 | -5.975 | -6.188 | -5.504 |
| 73 | -5.748 | -5.803 | -5.808 | -5.400 | -5.494 |
| 74 | -6.382 | -6.742 | -6.776 | -6.273 | -6.242 |
| 75 | -9.528 | - | - | - | - |
| 76 | - | -9.764 | -8.997 | - | - |
| 77 | - | - | - | -9.951 | -9.361 |
